# Acute promyelocytic leukemia: where did we start, where are we now, and the future

**DOI:** 10.1038/bcj.2015.25

**Published:** 2015-04-17

**Authors:** C C Coombs, M Tavakkoli, M S Tallman

**Affiliations:** 1 Memorial Sloan Kettering Cancer Center, Leukemia Service, New York, NY, USA; 2 Weill Cornell Medical College, New York, NY, USA

## Abstract

Historically, acute promyelocytic leukemia (APL) was considered to be one of the most fatal forms of acute leukemia with poor outcomes before the introduction of the vitamin A derivative all-*trans* retinoic acid (ATRA). With considerable advances in therapy, including the introduction of ATRA initially as a single agent and then in combination with anthracyclines, and more recently by development of arsenic trioxide (ATO)-containing regimens, APL is now characterized by complete remission rates of 90% and cure rates of ∼80%, even higher among low-risk patients. Furthermore, with ATRA–ATO combinations, chemotherapy may safely be omitted in low-risk patients. The disease is now considered to be the most curable subtype of acute myeloid leukemia (AML) in adults. Nevertheless, APL remains associated with a significant incidence of early death related to the characteristic bleeding diathesis. Early death, rather than resistant disease so common in all other subtypes of AML, has emerged as the major cause of treatment failure.

## Introduction

Acute promyelocytic leukemia (APL) is a unique subtype of acute myeloid leukemia (AML), with the first description as a distinct entity in 1957.^1^ The disease is identified by distinctive morphology and is distinguished by a balanced reciprocal translocation between chromosomes 15 and 17. Historically, APL has been characterized by a rapidly fatal course with a high incidence of early hemorrhagic death. This became evident in early studies when patients who were untreated or received corticosteroids experienced a median survival of <1 week, ranging from 1 day to 1 month.^2, 3, 4, 5, 6^ Current recommendations are that when a diagnosis of APL is suspected based upon clinical presentation and/or morphology, the disease should be treated as a medical emergency. Urgent administration of ATRA should be initiated with aggressive supportive measures including blood product support with platelets and cryoprecipitate while the genetic diagnosis is rapidly established.^7^

Risk stratification is imperative in the treatment of APL patients, as those with low-risk disease (white blood cell count (WBC) ≤10 000/μl) are generally treated with less intensive regimens than those patients presenting with high-risk disease (WBC >10 000/μl). Sanz *et al.*^8^ initially defined patients with WBC ≤10 000/μl and platelet count >40 000/μl as low risk for relapse, WBC ≤10 000/μl and platelet count ≤40k as intermediate risk and WBC >10 000/μl as high risk. However, as the outcomes for patients with low- and intermediate-risk disease are similar, these categories have been collapsed into one and considered as low-risk disease. In the past two decades, therapy for newly diagnosed APL has evolved from an all-*trans* retinoic acid (ATRA)+chemotherapy backbone for all patients to the addition of arsenic trioxide (ATO) to ATRA with omission of chemotherapy in low-risk patients as a new standard of care.

## Where did we start

### Induction regimens

APL has been associated with a high incidence of early hemorrhagic death. Early studies with induction including 6-mercatopurine (6-MP) alone or in combination with steroids, methyl-glyoxal guanyl hydrazine and/or methotrexate led to poor results.^9^ In the largest studies, remission rates were 5–14%, with survival ranging from 3 to 16 weeks (median 3.5 weeks) among all patients, and 4 months to >6 years among responders.^9, 10, 11, 12, 13, 14^ Despite waning beliefs that a cure could be attained, by the 1970s, anthracyclines were shown to yield complete remission (CR) rates that were at least comparable to, if not better than, those of other AML subtypes.^9, 15, 16, 17^

In 1973, daunorubicin (DNR) was shown to increase remission rates from 13 to 58% and to reduce hemorrhage-related mortality after 5 days of therapy relative to 6-MP-based regimens.^9^ It was also shown to induce durable remissions (median 26 months).^9^ Numerous investigators subsequently validated the efficacy of DNR in APL.^14, 15, 16, 17, 18, 19, 20, 21, 22, 23, 24, 25, 26^ Exceptional outcomes were later reported with higher dosing regimens (61% survival at 9 years, no relapses after 3 years).^22^ In addition, lower rates of death (41% vs 76%) and relapse (10% vs 68%) were reported in patients <50 years of age with increasing DNR doses (180–210 vs 40–135 mg/m^2^).^22^

Given the efficacy of DNR as a single agent, investigators sought to identify the superiority of anthracycline drug combinations over DNR alone. DNR was reported to yield similar rates of CR (67% vs 58%, *P* not significant) and early hemorrhagic death (10% vs 9%, *P* not significant) compared with various DNR and doxorubicin drug combinations in an analysis of 268 patients, although this analysis is limited by small numbers and its retrospective nature.^20^ The Southwest Oncology Group showed similar patient outcomes even with the addition of other chemotherapeutic agents to DNR.^22^ Despite similar rates of CR (73%) and death during induction (27%), Petti *et al.*^18^ reported more rapid responses (23 vs 45 days), longer durations of remission (14 vs 7 months) and better survival rates (27%, >6 years vs 0% >25 months) with DNR as a single agent relative to DNR-based drug combinations, limiting enthusiasm for combination therapy in these early studies.

### Pre-ATRA era reinduction, consolidation and maintenance therapy

Several early studies attempted to optimize reinduction for relapsed patients, consolidation and maintenance strategies in APL. Initial reports addressing outcomes following relapse were poor, with the best outcomes being achieved by Kantarjian *et al.*^21^ who reported a second CR rate (CR2) of 53% utilizing various reinduction regimens including combinations of doxorubicin, cytarabine, vincristine, amsacrine and prednisone. Cunningham *et al.*^15^ reported a median survival of 6 weeks following relapse. A variety of reinduction attempts were utilized in the pre-ATRA era; strategies including previous induction regimens were rarely successful.^15, 27^ Allogeneic hematopoietic stem cell transplantation (HSCT) yielded poor results during first CR (CR1); however, allogeneic and autologous HSCT resulted in the longest CR2 durations (29 to 48+ months).^15, 21, 27^ Furthermore, unlike other subtypes of AML, it had been recognized in the pre-ATRA era that specific maintenance regimens were shown to be critical to long-term survival. Of the patients, 42% receiving POMP (6-MP, methotrexate, vincristine and prednisone) maintenance were long-term survivors compared with 3% of those receiving cycling monthly chemotherapy.^28^ Kantarjian *et al.*^21^ also observed reduced remission durations when POMP maintenance was not used,^15, 21^ leading to support for maintenance regimens in future studies.

## Where we are now

### Induction regimens utilizing chemotherapy: remain the standard of care in high-risk APL

ATRA was introduced clinically in 1985, and this opened a new era in the treatment of APL.^29^ ATRA induces differentiation of leukemic promyelocytes into mature granulocytes, leading to its evaluation either as a single agent or in combination with chemotherapy, first in relapsed/refractory disease and then in newly diagnosed patients.^30, 31, 32, 33^ As a single agent, ATRA induced CR rates of 85% in studies by the Shanghai group in 1988.^33^ The first North American Intergroup study (I0129) demonstrated a 72% CR rate with single-agent ATRA, equivalent to rates obtained with conventional doses of cytarabine and DNR.^30^ However, frequent relapses were noted in patients who received ATRA alone. Continuous treatment with ATRA is characterized by reduction of its plasma concentration because of accelerated clearance.^29^ These findings prompted subsequent trials to combine ATRA with chemotherapy, leading to lower relapse rates.

Numerous prospective randomized studies were conducted to exploit the potential benefits of the combination of ATRA and chemotherapy. The European APL group demonstrated in a randomized study that concurrent ATRA plus chemotherapy (DNR and cytarabine) resulted in a lower relapse rate at 2 years (6% vs 16%, *P*=0.04)^34^ when compared with sequential ATRA followed by chemotherapy, and this has been confirmed in other large multicenter trials.^35, 36, 37, 38, 39^ Furthermore, the early addition of chemotherapy to ATRA decreased the incidence of retinoic acid syndrome.^40^ Ultimately, these studies established concurrent ATRA and anthracycline-based chemotherapy (either an anthracycline plus cytarabine or an anthracycline alone) as the standard of care for induction in newly diagnosed APL patients.

There has been controversy surrounding the optimal chemotherapy regimen to combine with ATRA. First, there are no definitive data to suggest the superiority of one anthracycline over another, as no prospective studies have been conducted comparing idarubicin with DNR in APL. Furthermore, there is no clear consensus on the role of cytarabine during induction therapy, although a number of studies have indicated that cytarabine is not needed in induction in any risk subset of patients. Two randomized trials investigated the role of cytarabine combined with either idarubicin or DNR, but yielded conflicting results.^41, 42^ The National Cancer Research Institute (NCRI) in the United Kingdom randomized patients between ATRA plus idarubicin (AIDA) and ATRA plus DNR and cytarabine (MRC AML15 trial), and reported no differences in response, relapse or overall survival (OS) rates, but less myelosuppression in the AIDA group.^42^ However, the study by the EuroAPL group (APL 2000) that randomized low-risk patients (age <60 and WBC ≤10 000/μl) to induction with ATRA/DNR/AraC versus ATRA/DNR and consolidation with DNR/AraC versus DNR reported an increase in 5-year cumulative incidence of relapse (CIR) (13.4% vs 29%, *P*=0.01) and a decrease in OS rates (92.9% vs 83.3%, *P*=0.07) in the group who did not receive cytarabine for induction and consolidation therapy.^41, 43^ Subsequent prospective, nonrandomized studies by the Gruppo Italiano Malattie Ematologiche dell'Adulto (GIMEMA) and Programa Español de Tratamientos en Hematología (PETHEMA) demonstrated that AIDA is as effective in inducing remission as cytarabine-containing regimens, with CR rates between 89 and 95%.^38, 44^ Differences in outcomes may be related to variation of individual studies, such as the consolidation regimens (ATRA vs no ATRA), the number of consolidation courses and the specific anthracycline used.

Given the favorable results from risk-adapted treatment strategies, first in the LPA99 trial followed by the LPA2005 trial, an additional induction option includes ATRA plus idarubicin with risk-adapted consolidation.^45, 46^ Finally, with the favorable results of the APML4 trial (discussed further below), which does not include cytarabine in induction (or consolidation), an alternate approach now recommended by the National Comprehensive Cancer Network (NCCN) includes ATRA plus idarubicin and ATO.^47^

### The introduction of ATO into the treatment of patients with APL

**Figure 1:**
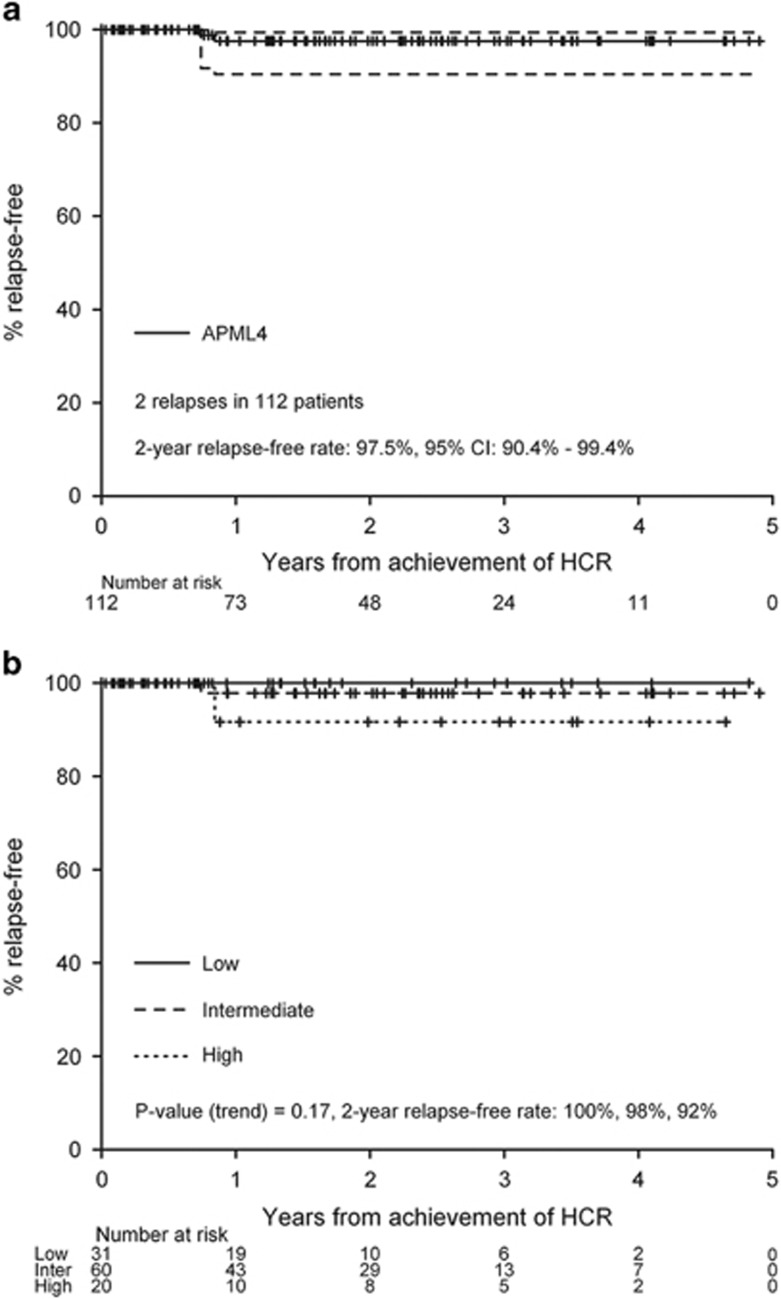
Relapse-free survival curves for APML4, the phase 2 trial utilizing combination of ATRA, ATO, and idarubicin in newly diagnosed APL. Panel **a** comprises all patients on APML4 (*n*=112) and panel **b** stratifies patients by Sanz risk category.

ATO was first utilized in APL patients in the early 1990s, and led to a high CR rate with relatively long-term remissions when used as a single agent.^48^ In preclinical models, the combination of ATRA and ATO demonstrated synergism in inducing differentiation and apoptosis,^49, 50, 51^ allowing for targeted therapy of APL without exposure to chemotherapy. This synergism between ATRA and ATO has been demonstrated to eradicate APL-initiating cells through promyelocytic leukemia/retinoic acid receptor-α degradation.^52^ Investigators at the Shanghai Institute of Hematology performed a randomized clinical trial in which patients received ATRA, ATO or the combination of ATRA plus ATO as induction therapy. Similar CR rates between groups (between 90 and 95.2%) were observed, but among the patients receiving combination ATRA–ATO therapy, there was a statistically significant improvement in the time to achieve CR, time for platelet recovery and decrease in the rate of relapse.^53^ The Australasian Leukaemia and Lymphoma Group (ALLG) performed a phase 2, single-armed study (APML4), reporting the outcome of 124 patients with newly diagnosed APL (23 patients with high-risk disease) treated with triple induction with ATRA, ATO and idarubicin, followed by two courses of consolidation with ATRA and ATO and 2 years of maintenance with ATRA, methotrexate and 6-MP (Figure 1).^47^ Outcomes were compared with historical controls from the APML3 study that used AIDA in induction and consolidation without ATO. With a median follow-up of 2 years, the 3-year OS and event-free survival (EFS) rates were 93.2% and 88.1%, respectively. Compared with APML3 results, this trial demonstrated a statistically significant improvement in freedom from relapse, disease-free survival (DFS) and failure-free survival, but not OS (Figure 1).^47^ Updated results with median follow-up of 4.2 years were reported at the 2014 meeting of the American Society of Hematology, with 5-year OS and EFS rates of 94% and 90%, respectively, in all risk groups (87% and 83% in high-risk patients, respectively).^54^ This regimen appears very promising; although, given its phase 2 nature and comparison with historical controls, it may be premature to suggest superiority. Furthermore, given the small number of high-risk patients, dedicated randomized trials in high-risk patients are required before drawing firm conclusions regarding the optimal induction regimen in this subset of patients.

Investigators at the MD Anderson Cancer Center demonstrated that the combination treatment of ATRA and ATO is an effective treatment in untreated APL with a high CR rate of 96%.^55^ However, high-risk patients (WBC >10 000/μl at presentation) achieved an inferior CR rate of 79–81% because of early treatment failure from fatal hemorrhage and differentiation syndrome despite the addition of either gemtuzumab ozogamicin (GO) or idarubicin during induction to control elevated WBC counts.^55, 56^ This suggests this regimen may be inadequate for high-risk patients.

In summary, these studies suggested that the combination of ATRA and ATO particularly in patients with low-risk disease is very promising. However, in patients presenting with high WBC, simultaneous use of cytotoxic agents such as anthracyclines in induction appears to be important to prevent rapid development of leukocytosis, differentiation syndrome and relapse, with a possible benefit of cytarabine in consolidation, discussed further below.

### The transition to nonchemotherapy-based approaches for low-risk disease: ATRA and ATO combination therapy

With the early success of ATRA- and ATO-based induction regimens, the question emerged as to whether chemotherapy could safely be eliminated or minimized to reduce treatment-associated toxicities and long-term complications observed with cytotoxic agents.^57^ This effort may be particularly important as therapy-related myeloid neoplasms have been observed in APL patients.^58, 59, 60^ In a recent series of 918 APL patients in CR, the incidence of therapy-related myeloid neoplasms was 2.2%, with the highest incidence of 5.2% in low-risk patients.^61^ The median OS from time of therapy-related myeloid neoplasm diagnosis in this series was 10 months; therefore, the omission of potentially leukemogenic cytotoxic chemotherapy is an attractive option to attempt to reduce the incidence of this serious complication.

**Figure 2:**
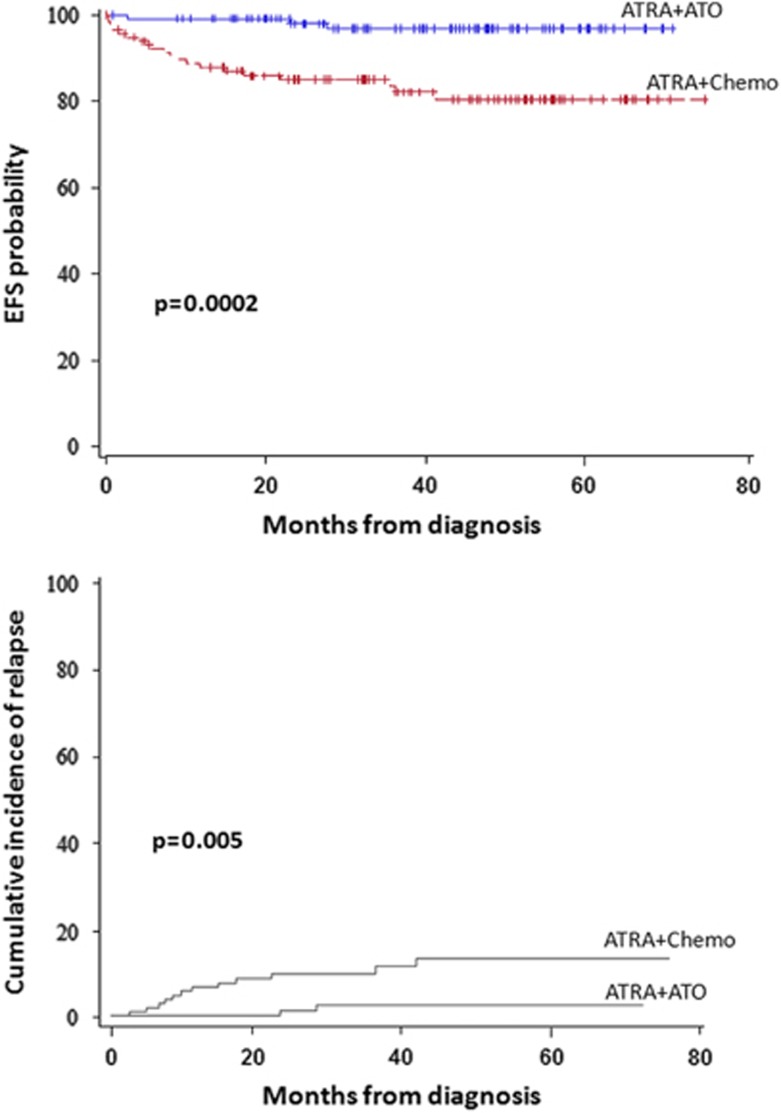
EFS probability and CIR in non-high-risk APL patients on Italian–German APL 0406 trial comparing ATRA–ATO with ATRA–chemotherapy on the extended final series.^62^

Given the success of single-center studies examining the combination of ATRA with ATO as described above, a phase 3, multicenter trial comparing ATRA plus idarubicin with ATRA plus ATO was conducted in patients with low- to intermediate-risk APL. In July 2013, Lo-Coco and colleagues^62^ published results of this trial, with average follow-up of 33.4 months with extended results of the final series of 276 patients presented at the 2014 American Society of Hematology meeting. The study was designed as a noninferiority trial to demonstrate that the rate of EFS between the groups was not >5%. The 2—year EFS rates were 97% in the ATRA–ATO group, and 86% in the ATRA–chemotherapy group meeting a *P*<0.001 for noninferiority and a *P*=0.02 for superiority, with EFS 98% vs 85% on updated series (*P*=0.0002) (Figure 2).^62^ The 2-year OS probability was 99% in the ATRA–ATO group, as compared with 91% in the ATRA–chemotherapy group (*P*=0.02). The 2-year DFS was 97% in the ATRA–ATO group and 90% in the ATRA–chemotherapy group (*P*=0.11), and the 2-year CIR was 1% in the ATRA–ATO group and 6% in the ATRA–chemotherapy group (CIR remained 1% in ATRA-ATO but increased to 9.4% for ATRA–chemotherapy in the updated analysis) (*P*=0.24 on initial analysis^63^ and *P*=0.005 in the updated analysis) (Figure 2).^62^ Toxicities differed between the two arms, in that hematologic toxicity occurred more frequently in the ATRA–chemotherapy arm, but hepatic toxicity and prolongation of the QTc interval occurred more frequently in the ATRA–ATO arm. Importantly, there was no difference in the incidence of differentiation syndrome between the arms, possibly related to the use of prophylactic prednisone in both groups.^63^ Health-related quality of life for fatigue severity was statistically improved in the ATRA–ATO arm as compared with ATRA–chemotherapy.^64^

In summary, ATRA–ATO was noninferior and possibly superior to ATRA–chemotherapy. The observed improvement in EFS and OS in the ATRA–ATO arm without significant differences in DFS and CIR suggests that these regimens have similar antileukemic efficacy, but with lower mortality in the ATRA–ATO arm from causes other than relapse.^63^ Longer-term follow-up will be important to draw final conclusions regarding efficacy and long-term toxicity.

Eghtedar *et al.*^65^ recently examined the incidence of secondary malignancies in patients treated with ATRA–ATO (*n*=106, with median follow-up of 29 months) versus ATRA–idarubicin (*n*=54, with median follow-up of 136 months). Nine patients in the chemotherapy group developed secondary malignancies compared with two patients in the ATRA–ATO group. They concluded that the treatment of APL patients using ATRA–ATO is not associated with a higher incidence of secondary malignancies with a *P*=0.29, adjusted for unit of time exposure. Longer follow-up of randomized populations such as the phase 3 study by Lo-Coco *et al.*^63^ would provide more useful estimations regarding long-term toxicities such as secondary malignancies.

Based upon these favorable results of the phase 3 trial comparing ATRA–ATO with ATRA–chemotherapy, ATRA–ATO has emerged as the new standard of care for patients with low-(to-intermediate) risk APL. Furthermore, ATRA–ATO therapy also may serve as an attractive alternative for patients who are considered unfit for conventional treatment and with severe comorbidities, such as older adults and patients with cardiac dysfunction or other severe organ dysfunction.

### Consolidation therapy: risk-adapted approach

Historical comparisons of trials by the GIMEMA^66^ and PETHEMA^45^ have demonstrated a lower relapse rate (8.7% vs 20.1%) and higher DFS and OS rates with concomitant administration of ATRA with chemotherapy in consolidation. However, no randomized studies have demonstrated this benefit of ATRA. Nevertheless, this approach has been routinely adopted.

There is no consensus regarding which specific chemotherapy is optimal in consolidation. The focus of past research efforts has been to develop risk-adapted strategies to provide more intensive treatment in high-risk patients with WBC >10 000/μl while minimizing toxicities in low-risk patients. A cooperative group multicenter study by PETHEMA (LPA2005) administered cytarabine only in high-risk patients, achieving a lower CIR at 3 years (11% vs 26%, *P*=0.03) compared with historical controls from LPA99 trial.^46^ Similarly, GIMEMA (AIDA2000) administered cytarabine in high-risk patients only and reported an improved incidence of relapse at 6 years in this group (9.3% vs 49.7%, *P*<0.001) compared with historical controls (AIDA0493).^66^ However, the improved outcome observed in the GIMEMA study is likely related to the use of ATRA in consolidation as the historical comparator received chemotherapy without ATRA. In contrast, a study by the NCRI, published only in abstract form, demonstrated no benefit of cytarabine in all risk groups of patients.^42^ Taken together, the majority of studies suggest a benefit of cytarabine in high-risk patients, possibly because of the synergistic effect of the combination of ATRA plus cytarabine.^67^ However, taking contemporary studies utilizing ATRA–ATO combination into account, it appears that cytarabine can be omitted in low-risk patients in consolidation and excellent outcome is preserved.^63^

To reduce chemotherapy exposure in low-risk patients, multiple cooperative groups have investigated the role of ATRA and ATO in consolidation. The North American Intergroup trial (C9710) randomized patients to receive two cycles of consolidation with ATRA plus DNR, either immediately following induction therapy or preceded by two 25-day cycles of ATO.^68^ The results demonstrated that for all risk groups, ATO in consolidation significantly improved 3-year DFS (90% vs 70%, *P*=<0.0001); and there was a nonstatistically significant improvement in OS (86% vs 81%, *P*=0.07).^68^ In a phase 2 study, Gore *et al.*^69^ reported comparable outcomes (DFS 90 and OS 88%) with considerably reduced amount of anthracyclines combined with a single cycle of ATO. Other groups have completely eliminated cytotoxic chemotherapy and investigated the role of ATO either as a single agent or combined with ATRA in consolidation. Using ATRA–ATO, with GO as alternate therapy for patients with toxicity to ATRA–ATO, investigators at the MD Anderson Cancer Center reported a 3-year OS of 85%.^70^ The ALLG reported a 3-year OS and EFS rates of 93% and 87%, respectively, utilizing ATRA–ATO in consolidation in APML4.^47^ Finally, the phase 3 trial by Lo Coco *et al.*^63^ demonstrated the utility of ATRA–ATO in consolidation for standard-risk patients, yielding at a minimum noninferior, and possibly superior, outcomes, as outlined above.

### Maintenance therapy

Prolonged maintenance therapy is typically included in modern APL treatment protocols, although its importance remains controversial. A Cochrane review examining published, ongoing and unpublished clinical trials through July 2012 sought to determine the role for maintenance therapy in APL in CR1. Selection criteria required randomized controlled trials assessing maintenance treatment in patients with newly diagnosed APL in CR1 following induction or induction and consolidation. Ten randomized trials enrolling 2072 patients were included in the systematic review, and meta-analysis was conducted on nine of these trials. There was no statistically significant improvement in OS in the comparisons examined (maintenance treatment vs observation, ATRA maintenance vs non-ATRA maintenance, ATRA maintenance alone vs ATRA with chemotherapy maintenance).^71^ However, DFS was improved with any maintenance compared with observation (hazard ratio 0.59, 95% confidence interval 0.48–0.74 with 1209 patients in 5 trials), although DFS was not statistically improved with ATRA-based regimens compared with non-ATRA regimens (hazard ratio 0.72, 95% confidence interval 0.51–1.01 with 670 patients from 4 trials).^71^ Although suggestive that maintenance may improve DFS, though not OS, in APL, the significant heterogeneity with regard to specific induction and consolidation regimens between these trials limits the generalized applicability of these findings.

Coutre *et al.*^72^ recently reported the results of the trial S0521 that randomized low-risk patients who achieved a molecular CR to maintenance with ATRA, 6-MP and methotrexate vs observation; all patients received standard induction of DNR, ATRA and cytarabine and consolidation with two courses each of ATO and DNR/cytarabine. Enrollment was stopped because of slow accrual. However, of the 68 patients randomized, no relapses were observed at median follow-up of 36.1 months, suggesting that in patients receiving intensive induction/consolidation including ATO, maintenance may not be necessary.

### Relapsed-refractory APL

With modern therapy, relapsed/refractory APL is a rare condition, as 90% of patients achieve CR after initial therapy and 80% of patients are cured of their disease. Delayed CR (that is, CR after 35 days of therapy) has been associated with a higher rate of relapse (31% vs 17%, *P*=0.001).^73^ Failure to achieve remission after ATRA-based induction therapy is rare, largely restricted to rare patients with ATRA-resistant variants, such as *PLZF-RARA-*positive APL.^74^ Resistance to ATO has recently been described in a series of 13 ATO-resistant APL patients using direct sequencing, 9 of whom harbored *PML* mutations, and 7 of these simultaneously harbored *RARA* mutations.^75^

Relapse occurs in 5–20% of patients, with <3% of patients with low-risk disease relapsing, but closer to 20% relapse rate in some series among high-risk patients, although this rate appears to be lower at ∼10–12% in contemporary series.^47, 76^ Relapse at extramedullary sites is an increasingly recognized problem, occurring in 3–5% of patients.^77^ Therapeutic options for relapsed/refractory APL have included ATO, thought to be the single most active agent in APL, with 40 of 47 relapsed APL patients achieving CR in an early study.^48^ Further treatment options for induction include combinations of ATO with chemotherapy such as anthracyclines and anti-CD33 humanized antibodies (discussed further below).

However, as ATO moves to front-line therapeutic strategies, the response to ATO in relapse to those patients previously exposed to ATO is unclear. This issue will become an important one, although for increasingly fewer patients. A retrospective study examined 64 consecutive first-relapsed APL patients receiving salvage therapy with ATO and chemotherapy, 52 of whom had a hematologic relapse. Of patients with hematologic relapse, 20 had relapsed after previous ATO therapy and 32 did not receive prior ATO therapy.^78^ There was no statistical difference between CR2 rate (80% vs 93.8%, *P*=0.189) or 4-year OS rate (62.4% vs 71.2%, *P*=0.816), but there was a statistically significant difference between relapse rate (68.8% vs 33.3%, *P*=0.03) and 4-year relapse-free survival rate (29.8% vs 66.2%, *P*=0.023).^78^ This study is limited by its retrospective design and small number of patients. Larger prospective studies may help elucidate the utility of rechallenge with ATO in previously exposed patients.

Once a patient has achieved CR2, HSCT is considered in patients who are candidates. Autologous as well as allogeneic transplants have been evaluated.^79^ Although both have been associated with durable remission and prolonged survival, the former approach has led to the best outcomes in all comparative studies. A phase 2 study of 35 patients evaluating the efficacy and feasibility of induction and consolidation with ATO followed by auto-HSCT in relapsed APL demonstrated a 5-year EFS of 65% and a 5-year OS of 77%.^80^ Recent data suggest an improved 5-year DFS and OS in auto-HSCT when compared with allo-HSCT (DFS 63% in auto-HSCT and 50% in allo-HSCT (*P*=0.10); OS 75% in auto-HSCT vs 54% in allogeneic (*P*=0.002)).^81^ In a retrospective study that reviewed patients who received ATO-based therapy before auto-HSCT, a delay in neutrophil recovery has been demonstrated, although the clinical significance is uncertain.^82^ Owing to the increasing use of ATO in front-line therapy for APL, larger prospective studies are necessary to validate such findings and to understand the mechanism of delayed neutrophil recovery.^82^

## The future

### Efforts to improve early death rate

Unlike other subtypes of AML, the primary cause of treatment failure in patients with APL is early death, defined as death within the first 30 days of diagnosis. Although the rate of early death is low in patients enrolled on clinical trials, it is significantly higher in patients who are not enrolled on trials, likely related to selection bias (20–30% compared with 3% in a recent study^83^). Early death is particularly common in older patients.^84^ The observed improvement in early death rate over time is modest at best, decreasing from 22.1% in 1992–1995, to 14.7% in 1996–2001 and 17.5% between 2002 and 2007, in a population-based study.^85^ The reasons for early deaths in APL are multiple, although death during induction is most frequently related to the hemorrhagic diathesis because of hyperfibrinolysis, proteolysis and disseminated intravascular coagulation, further complicated by thrombocytopenia.^86^ Delays in ATRA therapy have been suggested as a contributing factor in early deaths, with ATRA ordered in only 31% of APL on the day the diagnosis was suspected in one retrospective analysis.^87^ In another retrospective review examining early APL deaths, delay in ATRA administration was not a statistically significant cause for early death, although interpretation of these data is limited as the group with delayed ATRA therapy was generally less sick than the group that received ATRA promptly.^88^

Ultimately, given excellent response rates in APL with low relapse rates even among high-risk patients, improvement in the early death rate in APL is of paramount importance. Education of medical providers should lead to a high level of vigilance regarding this diagnosis, to facilitate prompt suspicion for the diagnosis of APL, at which time ATRA should be initiated in addition to aggressive supportive measures. There is general consensus regarding aggressive blood product support, in that platelets should be maintained above 30–50 × 10^9^/l and fibrinogen above 100–150 mg/dl.^89^

### Novel agents

#### Oral ATO

**Figure 3:**
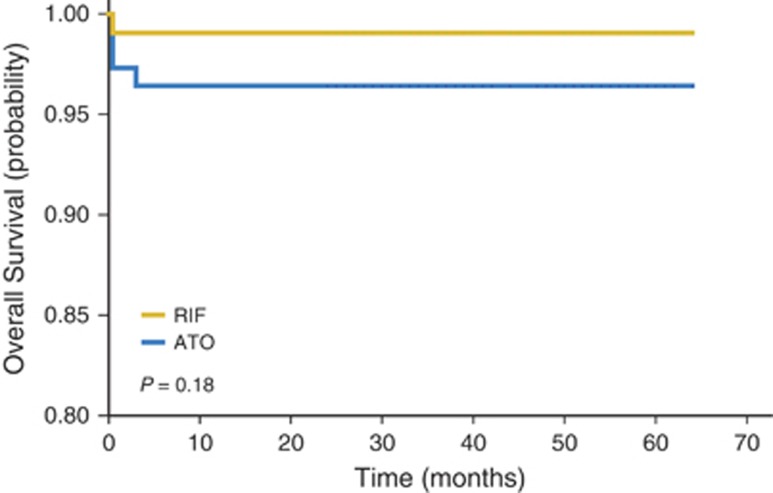
Overall survival curves for phase 3 randomized noninferiority trial comparing oral ATO (realgar-indigo naturalis formula (RIF) with i.v. ATO.^95^

In the aforementioned studies examining therapy with ATO, the intravenous (i.v.) formulation was utilized. The use of i.v. ATO is inconvenient, as it requires frequent patient visits for administration and maintenance of vascular access, further complicated by an observed increase in the rate of central venous catheter-associated thrombosis among APL patients compared with acute lymphocytic leukemia and AML patients.^90^ An oral formulation of ATO has been developed that showed favorable oral absorption with an achieved bioavailability of up to 95% of an equivalent dose of i.v. ATO.^91^ Oral ATO was first utilized in the treatment of relapsed APL that showed high efficacy and similar toxicity profile to i.v. formulations.^92^ Notably, the QTc prolongation and ventricular arrhythmias seen with i.v. ATO were not observed with oral ATO, likely because of lower peak plasma arsenic concentrations achieved with oral formulations.^93^ Oral ATO has since been tested in the setting of maintenance after first CR, and with 10-year follow-up, this regimen appears to have similar outcomes to i.v. formulations.^94^ Finally, oral ATO versus i.v. ATO in combination with ATRA was examined in a randomized, phase 3 noninferiority trial, and oral ATO with ATRA was noninferior to i.v. ATO with ATRA (Figure 3).^95^ In summary, the oral formulation of ATO exhibit excellent activity and combinations with ATRA provide an opportunity for a completely oral, chemotherapy-free regimen for treating APL.^96^ Although oral ATO is an attractive therapeutic approach, longer-term follow-up is needed, and it is not yet readily available in the United States.

#### Anti-CD33 monoclonal antibodies

GO is an anti-CD33 monoclonal antibody conjugated to the toxin calicheamicin, and has shown significant activity in APL because of the high level of expression of CD33 target antigen on APL cells.^97^ However, safety concerns led to the US marketing withdrawal of GO in June 2010, although this decision has more recently been called into question.^98, 99^ GO is currently available under compassionate use programs. SGN-CD33A is a next-generation anti-CD33 antibody currently in clinical trials for AML, including APL, that has demonstrated antileukemic activity with 47% blast clearance in interim analysis of the phase 1 study.^100^

Lo-Coco *et al.*^101^ explored the use of GO as a single agent in relapsed APL. Of the 16 patients treated, a molecular remission was obtained in 11 patients after two doses, and in an additional two patients after the third dose. One patient achieved molecular remission after first dose but was taken off drug because of hepatic toxicity. The last two patients had disease progression during treatment. These results supported that GO has significant single-agent activity in relapsed APL.^101^

Ravandi *et al.*^70^ published the MD Anderson experience of utilizing ATRA–ATO induction with the addition of GO in high-risk, newly diagnosed APL patients (WBC ≥10 000/μl at presentation in all patients, or WBC >30 000/μl during induction in the second cohort of patients). Post-remission therapy consisted of ATRA and ATO, with GO given if either ATRA or ATO were discontinued because of toxicity. In the 82 patients examined, 74 achieved a CR with one additional CR with incomplete platelet recovery. The CR rate for low-risk patients was 95% and CR rate in high-risk patients was 81%.^70^

#### Tamibarotene

Tamibarotene (formerly called Am80) is a synthetic retinoid that induces differentiation of HL-60 and NB-4 cells with ∼10 times more potent *in vitro* activity compared with ATRA, with a favorable pharmacokinetic profile as the plasma level does not decline after daily administration.^102, 103^ A phase 3 study was conducted to compare tamibarotene with ATRA as maintenance therapy for patients with newly diagnosed APL. Of the 344 eligible patients, 319 (93%) achieved CR with 269 undergoing maintenance randomization after completing three courses of consolidation.^104^ There was no statistical difference between ATRA and tamibarotene for relapse-free survival, although in an exploratory analysis, high-risk patients were noted to have an improved relapse-free survival rate of 87% in the tamibarotene arm as compared with 58% in the ATRA arm.^104^ Tamibarotene was examined as a single agent for induction in relapsed/refractory APL, showing activity in patients who previously received ATRA and ATO; however, responses were not durable.^105^ Ultimately, the utility of this agent in the ATO era is of uncertain significance, although it possibly may have a role in high-risk patients for maintenance therapy, but this would needs to be confirmed in larger, dedicated studies.

### Survivorship

Given the exceedingly high cure rate with modern therapy and the relatively young median age of patients, a future focus should emphasize optimization of survivorship care for APL patients. In a recent series, outcomes for APL patients treated with ATRA–ATO and ATRA–chemotherapy who were in CR for at least 3 years were retrospectively examined, revealing an 8% incidence of second malignancies in addition to the development of comorbid conditions such as diabetes mellitus, hypertension and cardiac disease, emphasizing the importance of long-term follow-up for APL survivors.^106^

## Conclusion

APL has been transformed from the most fatal to the most curable form of acute leukemia in adults. The standard of care for low-risk patients no longer includes chemotherapy given the success of the phase 3 noninferiority trial examining ATRA–ATO combination therapy. Regimens for treating high-risk APL have not been sufficiently compared to suggest superiority of one regimen over another. Given the tolerability and excellent long-term outcomes, our approach for high-risk patients includes triple induction with ATRA, ATO and idarubicin. Areas of ongoing need include efforts to decrease the early death rate, which is the primary cause for treatment failure, refinements in strategies for high-risk patients and a focus on survivorship care.

APL has served as a paradigm for targeted, differentiation-based therapies, with ATRA and ATO changing the landscape of therapy for this once uniformly fatal disease.

## References

[bib1] Hillestad LK Acute promyelocytic leukemia Acta Med Scand 1957 159 189 194 13508085

[bib2] Pisciotta AV Schulz EJ Fibrinolytic purpura in acute leukemia Am J Med 1955 19 824 828 13268484 10.1016/s0002-9343(55)80028-9

[bib3] Cooperberg AA Neiman GM Fibrinogenopenia and fibrinolysis in acute myelogenous leukemia Ann Intern Med 1955 42 706 711 14350491 10.7326/0003-4819-42-3-706

[bib4] Fisher S Ramot B Kreisler B Fibrinolysis in Acute Leukemia Israel Med J 1960 XIX 195 198 13699997

[bib5] Ghitis J Acute promyelocytic leukemia Blood 1963 21 237 240 13947503

[bib6] Larson RA Kondo K Vardiman JW Butler AE Golomb HM Rowley JD Evidence for a 15;17 translocation in every patient with acute promyelocytic leukemia Am J Med 1984 76 827 841 6586073 10.1016/0002-9343(84)90994-x

[bib7] Sanz MA Grimwade D Tallman MS Lowenberg B Fenaux P Estey EH Management of acute promyelocytic leukemia: recommendations from an expert panel on behalf of the European LeukemiaNet Blood 2009 113 1875 1891 18812465 10.1182/blood-2008-04-150250

[bib8] Sanz MA Lo Coco F Martin G Avvisati G Rayon C Barbui T Definition of relapse risk and role of nonanthracycline drugs for consolidation in patients with acute promyelocytic leukemia: a joint study of the PETHEMA and GIMEMA cooperative groups Blood 2000 96 1247 1253 10942364

[bib9] Bernard J Weil M Boiron M Jacquillat C Flandrin G Gemon MF Acute promyelocytic leukemia: results of treatment by daunorubicin Blood 1973 41 489 496 4510926

[bib10] Bernard PJ Mathe G Boulay J Ceoard B Chome J La leucose aigue a promyelocytes Schweiz Med Wochenschr 1959 23 604 608 13799642

[bib11] Baker WG Bang NU Nachman RL Raafat F Horowitz HI Hypofibrinogenemic hemorrhage in acute myelogenous leukemia treated with heparin. With autopsy findings of widespread intravascular clotting Ann Intern Med 1964 61 116 123 14175831 10.7326/0003-4819-61-1-116

[bib12] Rosenthal RL Acute promyelocytic leukemia associated with hypofibrinogenemia Blood 1963 21 495 508 13974993

[bib13] Didisheim P Trombold JS Vandervoort LE Mibashan RS Acute promyelocytic leukemia with fibrinogen and factor V deficiencies Blood 1964 23 717 728 14161406

[bib14] Drapkin RL Gee TS Dowling MD Arlin Z McKenzie S Kempin S Prophylactic heparin therapy in acute promyelocytic leukemia Cancer 1978 41 2484 2490 274995 10.1002/1097-0142(197806)41:6<2484::aid-cncr2820410659>3.0.co;2-#

[bib15] Cunningham I Gee TS Reich LM Kempin SJ Naval AN Clarkson BD Acute promyelocytic leukemia: treatment results during a decade at Memorial Hospital Blood 1989 73 1116 1122 2930837

[bib16] Sanz MA Jarque I Martin G Lorenzo I Martinez J Rafecas J Acute promyelocytic leukemia. Therapy results and prognostic factors Cancer 1988 61 7 13 3422032 10.1002/1097-0142(19880101)61:1<7::aid-cncr2820610103>3.0.co;2-6

[bib17] Cordonnier C Vernant JP Brun B Heilmann MG Kuentz M Bierling P Acute promyelocytic leukemia in 57 previously untreated patients Cancer 1985 55 18 25 3855265 10.1002/1097-0142(19850101)55:1<18::aid-cncr2820550104>3.0.co;2-b

[bib18] Petti MC Avvisati G Amadori S Baccarani M Guarini AR Papa G Acute promyelocytic leukemia: clinical aspects and results of treatment in 62 patients Haematologica 1987 72 151 155 3114070

[bib19] Arlin Z Kempin S Mertelsmann R Gee T Higgins C Jhanwar S Primary therapy of acute promyelocytic leukemia: results of amsacrine- and daunorubicin-based therapy Blood 1984 63 211 212 6580926

[bib20] Rodeghiero F Avvisati G Castaman G Barbui T Mandelli F Early deaths and anti-hemorrhagic treatments in acute promyelocytic leukemia. A GIMEMA retrospective study in 268 consecutive patients Blood 1990 75 2112 2117 2189506

[bib21] Kantarjian HM Keating MJ Walters RS Estey EH McCredie KB Smith TL Acute promyelocytic leukemia Am J Med 1986 80 789 797 3458366 10.1016/0002-9343(86)90617-0

[bib22] Head D Kopecky KJ Weick J Files JC Ryan D Foucar K Effect of aggressive daunomycin therapy on survival in acute promyelocytic leukemia Blood 1995 86 1717 1728 7655004

[bib23] Goldberg MA Ginsburg D Mayer RJ Stone RM Maguire M Rosenthal DS Is heparin administration necessary during induction chemotherapy for patients with acute promyelocytic leukemia Blood 1987 69 187 191 3466655

[bib24] Collins AJ Bloomfield CD Peterson BA McKenna RW Edson JR Acute promyelocytic leukemia. Management of the coagulopathy during daunorubicin-prednisone remission induction Arch Intern Med 1978 138 1677 1680 281191 10.1001/archinte.138.11.1677

[bib25] Leone G Mango G Alfano G Disseminated intravascular coagulation in acute promyelocytic leukemia. Possibility of treatment with glucocorticoids at high doses Nouv Rev Fr Hematol 1978 20 395 401 289100

[bib26] Daly PA Schiffer CA Wiernik PH Acute promyelocytic leukemia-clinical management of 15 patients Am J Hematol 1980 8 347 359 6932177 10.1002/ajh.2830080403

[bib27] Avvisati G Petti MC Petrucci MT Falconi E Tirindelli MC Mandelli F Treatment of recurrent promyelocytic leukemia with a combination regimen utilizing amsacrine, cytosine arabinoside and 6-thioguanine (AAT) Haematologica 1989 74 279 282 2511097

[bib28] Marty M Ganem G Fischer J Flandrin G Berger R Schaison G [Acute promyelocytic leukemia: retrospective study of 119 patients treated with daunorubicin] Nouv Rev Fr Hematol 1984 26 371 378 6597407

[bib29] Wang ZY Chen Z Acute promyelocytic leukemia: from highly fatal to highly curable Blood 2008 111 2505 2515 18299451 10.1182/blood-2007-07-102798

[bib30] Tallman MS Andersen JW Schiffer CA Appelbaum FR Feusner JH Ogden A All-trans-retinoic acid in acute promyelocytic leukemia N Engl J Med 1997 337 1021 1028 9321529 10.1056/NEJM199710093371501

[bib31] Chen ZX Xue YQ Zhang R Tao RF Xia XM Li C A clinical and experimental study on all-trans retinoic acid-treated acute promyelocytic leukemia patients Blood 1991 78 1413 1419 1884013

[bib32] Castaigne S Chomienne C Daniel MT Ballerini P Berger R Fenaux P All-trans retinoic acid as a differentiation therapy for acute promyelocytic leukemia. I. Clinical results Blood 1990 76 1704 1709 2224119

[bib33] Huang ME Ye YC Chen SR Chai JR Lu JX Zhoa L Use of all-trans retinoic acid in the treatment of acute promyelocytic leukemia Blood 1988 72 567 572 3165295

[bib34] Fenaux P Chastang C Chevret S Sanz M Dombret H Archimbaud E A randomized comparison of all transretinoic acid (ATRA) followed by chemotherapy and ATRA plus chemotherapy and the role of maintenance therapy in newly diagnosed acute promyelocytic leukemia. The European APL Group Blood 1999 94 1192 1200 10438706

[bib35] Lengfelder E Reichert A Schoch C Haase D Haferlach T Loffler H Double induction strategy including high dose cytarabine in combination with all-trans retinoic acid: effects in patients with newly diagnosed acute promyelocytic leukemia. German AML Cooperative Group Leukemia 2000 14 1362 1370 10942230 10.1038/sj.leu.2401843

[bib36] Mandelli F Diverio D Avvisati G Luciano A Barbui T Bernasconi C Molecular remission in PML/RAR alpha-positive acute promyelocytic leukemia by combined all-trans retinoic acid and idarubicin (AIDA) therapy. Gruppo Italiano-Malattie Ematologiche Maligne dell'Adulto and Associazione Italiana di Ematologia ed Oncologia Pediatrica Cooperative Groups Blood 1997 90 1014 1021 9242531

[bib37] Asou N Adachi K Tamura J Kanamaru A Kageyama S Hiraoka A Analysis of prognostic factors in newly diagnosed acute promyelocytic leukemia treated with all-trans retinoic acid and chemotherapy. Japan Adult Leukemia Study Group J Clin Oncol 1998 16 78 85 9440726 10.1200/JCO.1998.16.1.78

[bib38] Sanz MA Martin G Rayon C Esteve J Gonzalez M Diaz-Mediavilla J A modified AIDA protocol with anthracycline-based consolidation results in high antileukemic efficacy and reduced toxicity in newly diagnosed PML/RARalpha-positive acute promyelocytic leukemia. PETHEMA group Blood 1999 94 3015 3021 10556184

[bib39] Fenaux P Le Deley MC Castaigne S Archimbaud E Chomienne C Link H Effect of all transretinoic acid in newly diagnosed acute promyelocytic leukemia. Results of a multicenter randomized trial. European APL 91 Group Blood 1993 82 3241 3249 8241496

[bib40] de Botton S Chevret S Coiteux V Dombret H Sanz M San Miguel J Early onset of chemotherapy can reduce the incidence of ATRA syndrome in newly diagnosed acute promyelocytic leukemia (APL) with low white blood cell counts: results from APL 93 trial Leukemia 2003 17 339 342 12592333 10.1038/sj.leu.2402807

[bib41] Ades L Chevret S Raffoux E de Botton S Guerci A Pigneux A Is cytarabine useful in the treatment of acute promyelocytic leukemia? Results of a randomized trial from the European Acute Promyelocytic Leukemia Group J Clin Oncol 2006 24 5703 5710 17116939 10.1200/JCO.2006.08.1596

[bib42] Burnett AK Hills RK Grimwade D Goldstone AH Hunter A Milligan D Idarubicin and ATRA is as effective as MRC chemotherapy in patients with acute promyelocytic leukaemia with lower toxicity and resource usage: preliminary results of the MRC AML15 Trial ASH Annual Meeting Abstracts. 2007 110 589

[bib43] Ades L Raffoux E Chevret S de Botton S Guerci A Pigneux A Is AraC required in the treatment of standard risk APL? Long term results of a randomized trial (APL 2000) from the French Belgian Swiss APL Group ASH Annual Meeting Abstracts 2010 116 13

[bib44] Avvisati G Lo Coco F Diverio D Falda M Ferrara F Lazzarino M AIDA (all-trans retinoic acid+idarubicin) in newly diagnosed acute promyelocytic leukemia: a Gruppo Italiano Malattie Ematologiche Maligne dell'Adulto (GIMEMA) pilot study Blood 1996 88 1390 1398 8695858

[bib45] Sanz MA Martin G Gonzalez M Leon A Rayon C Rivas C Risk-adapted treatment of acute promyelocytic leukemia with all-trans-retinoic acid and anthracycline monochemotherapy: a multicenter study by the PETHEMA group Blood 2004 103 1237 1243 14576047 10.1182/blood-2003-07-2462

[bib46] Sanz MA Montesinos P Rayon C Holowiecka A de la Serna J Milone G Risk-adapted treatment of acute promyelocytic leukemia based on all-trans retinoic acid and anthracycline with addition of cytarabine in consolidation therapy for high-risk patients: further improvements in treatment outcome Blood 2010 115 5137 5146 20393132 10.1182/blood-2010-01-266007

[bib47] Iland HJ Bradstock K Supple SG Catalano A Collins M Hertzberg M All-trans-retinoic acid, idarubicin, and IV arsenic trioxide as initial therapy in acute promyelocytic leukemia (APML4) Blood 2012 120 1570 1580quiz 752. 22715121 10.1182/blood-2012-02-410746

[bib48] Niu C Yan H Yu T Sun HP Liu JX Li XS Studies on treatment of acute promyelocytic leukemia with arsenic trioxide: remission induction, follow-up, and molecular monitoring in 11 newly diagnosed and 47 relapsed acute promyelocytic leukemia patients Blood 1999 94 3315 3324 10552940

[bib49] Zheng PZ Wang KK Zhang QY Huang QH Du YZ Zhang QH Systems analysis of transcriptome and proteome in retinoic acid/arsenic trioxide-induced cell differentiation/apoptosis of promyelocytic leukemia Proc Natl Acad Sci USA 2005 102 7653 7658 15894607 10.1073/pnas.0502825102 PMC1140456

[bib50] Gianni M Koken MH Chelbi-Alix MK Benoit G Lanotte M Chen Z Combined arsenic and retinoic acid treatment enhances differentiation and apoptosis in arsenic-resistant NB4 cells Blood 1998 91 4300 4310 9596679

[bib51] Dos Santos GA Kats L Pandolfi PP Synergy against PML-RARa: targeting transcription, proteolysis, differentiation, and self-renewal in acute promyelocytic leukemia J Exp Med 2013 210 2793 2802 24344243 10.1084/jem.20131121 PMC3865469

[bib52] Nasr R Guillemin MC Ferhi O Soilihi H Peres L Berthier C Eradication of acute promyelocytic leukemia-initiating cells through PML-RARA degradation Nat Med 2008 14 1333 1342 19029980 10.1038/nm.1891

[bib53] Shen ZX Shi ZZ Fang J Gu BW Li JM Zhu YM All-trans retinoic acid/As2O3 combination yields a high quality remission and survival in newly diagnosed acute promyelocytic leukemia Proc Natl Acad Sci USA 2004 101 5328 5335 15044693 10.1073/pnas.0400053101 PMC397380

[bib54] Iland HJ Collins M Hertzberg MS Seldon M Grigg AP Firkin FFinal analysis of the Australasian Leukaemia and Lymphoma Group (ALLG) APML4 Trial: all-trans retinoic acid (ATRA), intravenous arsenic trioxide (ATO) and idarubicin (IDA) as initial therapy for acute promyelocytic leukemia (APL). American Society of Hematology Meeting, San Francisco, CA, USA, 2014..

[bib55] Estey E Garcia-Manero G Ferrajoli A Faderl S Verstovsek S Jones D Use of all-trans retinoic acid plus arsenic trioxide as an alternative to chemotherapy in untreated acute promyelocytic leukemia Blood 2006 107 3469 3473 16373661 10.1182/blood-2005-10-4006

[bib56] Ravandi F Estey EH Cortes JE O‘Brien S Pierce SA Brandt M Phase II study of all-trans retinoic acid (ATRA), arsenic trioxide (ATO), with or without gemtuzumab ozogamicin (GO) for the frontline therapy of patients with acute promyelocytic leukemia (APL) Blood (ASH Annual Meeting Abstracts) 2010 116 Abstract 1080

[bib57] Pellicori P Calicchia A Lococo F Cimino G Torromeo C Subclinical anthracycline cardiotoxicity in patients with acute promyelocytic leukemia in long-term remission after the AIDA protocol Congest Heart Fail (Greenwich, Conn) 2012 18 217 221 10.1111/j.1751-7133.2011.00278.x 22809260

[bib58] Andersen MK Pedersen-Bjergaard J Therapy-related MDS and AML in acute promyelocytic leukemia Blood 2002 100 1928 1929author reply 9. 12211197 10.1182/blood-2002-03-0962

[bib59] Garcia-Manero G Kantarjian HM Kornblau S Estey E Therapy-related myelodysplastic syndrome or acute myelogenous leukemia in patients with acute promyelocytic leukemia (APL) Leukemia 2002 16 1888 12200720 10.1038/sj.leu.2402616

[bib60] Latagliata R Petti MC Fenu S Mancini M Spiriti MA Breccia M Therapy-related myelodysplastic syndrome-acute myelogenous leukemia in patients treated for acute promyelocytic leukemia: an emerging problem Blood 2002 99 822 824 11806982 10.1182/blood.v99.3.822

[bib61] Montesinos P Gonzalez JD Gonzalez J Rayon C de Lisa E Amigo ML Therapy-related myeloid neoplasms in patients with acute promyelocytic leukemia treated with all-trans-retinoic Acid and anthracycline-based chemotherapy J Clin Oncol 2010 28 3872 3879 20625122 10.1200/JCO.2010.29.2268

[bib62] Platzbecker U Avvisati G Ehninger G Cicconi L Thiede C Ferrara FImproved outcome with ATRA-arsenic trioxide compared to ATRA-chemotherapy in non-high risk acute promyelocytic leukemia – updated results of the Italian-German APL0406 Trial on the Extended Final Series. American Society of Hematology Meeting, San Francisco, CA, USA, 2014.

[bib63] Lo-Coco F Avvisati G Vignetti M Thiede C Orlando SM Iacobelli S Retinoic acid and arsenic trioxide for acute promyelocytic leukemia N Engl J Med 2013 369 111 121 23841729 10.1056/NEJMoa1300874

[bib64] Efficace F Mandelli F Avvisati G Cottone F Ferrara F Di Bona E Randomized phase III trial of retinoic acid and arsenic trioxide versus retinoic acid and chemotherapy in patients with acute promyelocytic leukemia: health-related quality-of-life outcomes J Clin Oncol 2014 32 3406 3412 25245446 10.1200/JCO.2014.55.3453

[bib65] Eghtedar A Rodriguez I Kantarjian H O'Brien S Daver N Garcia-Manero G Incidence of secondary neoplasms in patients with acute promyelocytic leukemia treated with all-trans retinoic acid plus chemotherapy or with all-trans retinoic acid plus arsenic trioxide Leuk Lymph 2014e-pub ahead of print 3 November 2014doi:10.3109/10428194.2014.953143 PMC4417657 25120050

[bib66] Lo-Coco F Avvisati G Vignetti M Breccia M Gallo E Rambaldi A Front-line treatment of acute promyelocytic leukemia with AIDA induction followed by risk-adapted consolidation for adults younger than 61 years: results of the AIDA-2000 trial of the GIMEMA Group Blood 2010 116 3171 3179 20644121 10.1182/blood-2010-03-276196

[bib67] Flanagan SA Meckling KA All- trans-retinoic acid increases cytotoxicity of 1-beta-D-arabinofuranosylcytosine in NB4 cells Cancer Chemother Pharmacol 2003 51 363 375 12736759 10.1007/s00280-002-0561-0

[bib68] Powell BL Moser B Stock W Gallagher RE Willman CL Stone RM Arsenic trioxide improves event-free and overall survival for adults with acute promyelocytic leukemia: North American Leukemia Intergroup Study C9710 Blood 2010 116 3751 3757 20705755 10.1182/blood-2010-02-269621 PMC2981533

[bib69] Gore SD Gojo I Sekeres MA Morris L Devetten M Jamieson K Single cycle of arsenic trioxide-based consolidation chemotherapy spares anthracycline exposure in the primary management of acute promyelocytic leukemia J Clin Oncol 2010 28 1047 1053 20085935 10.1200/JCO.2009.25.5158 PMC2834430

[bib70] Ravandi F Estey E Jones D Faderl S O'Brien S Fiorentino J Effective treatment of acute promyelocytic leukemia with all-trans-retinoic acid, arsenic trioxide, and gemtuzumab ozogamicin J Clin Oncol 2009 27 504 510 19075265 10.1200/JCO.2008.18.6130 PMC4881307

[bib71] Muchtar E Vidal L Ram R Gafter-Gvili A Shpilberg O Raanani P The role of maintenance therapy in acute promyelocytic leukemia in the first complete remission Cochrane Database Syst Rev 2013 3 Cd009594 23543579 10.1002/14651858.CD009594.pub2 PMC12127902

[bib72] Coutre SE Othus M Powell B Willman CL Stock W Paietta E Arsenic trioxide during consolidation for patients with previously untreated low/intermediate risk acute promyelocytic leukaemia may eliminate the need for maintenance therapy Br J Haematol 2014 165 497 503 24528179 10.1111/bjh.12775 PMC4064464

[bib73] Breccia M Minotti C Latagliata R Loglisci G Salaroli A Loglisci MG Influence of time to complete remission and duration of all-trans retinoic acid therapy on the relapse risk in patients with acute promyelocytic leukemia receiving AIDA protocols Leuk Res 2013 37 383 385 23259988 10.1016/j.leukres.2012.11.014

[bib74] Licht JD Chomienne C Goy A Chen A Scott AA Head DR Clinical and molecular characterization of a rare syndrome of acute promyelocytic leukemia associated with translocation (11;17) Blood 1995 85 1083 1094 7849296

[bib75] Zhu HH Qin YZ Huang XJ Resistance to arsenic therapy in acute promyelocytic leukemia N Engl J Med 2014 370 1864 1866 24806185 10.1056/NEJMc1316382

[bib76] Tallman MS Treatment of relapsed or refractory acute promyelocytic leukemia Best Pract Res Clin Haematol 2007 20 57 65 17336255 10.1016/j.beha.2006.11.002

[bib77] Specchia G Lo Coco F Vignetti M Avvisati G Fazi P Albano F Extramedullary involvement at relapse in acute promyelocytic leukemia patients treated or not with all-trans retinoic acid: a report by the Gruppo Italiano Malattie Ematologiche dell'Adulto J Clin Oncol 2001 19 4023 4028 11600603 10.1200/JCO.2001.19.20.4023

[bib78] Lou Y Suo S Tong Y Tong H Qian W Meng H Outcomes and prognostic factors of first relapsed acute promyelocytic leukemia patients undergoing salvage therapy with intravenous arsenic trioxide and chemotherapy Ann Hematol 2014 93 941 948 24408159 10.1007/s00277-013-2000-1

[bib79] Pemmaraju N Tanaka MF Ravandi F Lin H Baladandayuthapani V Rondon G Outcomes in patients with relapsed or refractory acute promyelocytic leukemia treated with or without autologous or allogeneic hematopoietic stem cell transplantation Clin Lymphoma Myeloma Leuk 2013 13 485 492 23769669 10.1016/j.clml.2013.02.023 PMC4112369

[bib80] Yanada M Tsuzuki M Fujita H Fujimaki K Fujisawa S Sunami K Phase 2 study of arsenic trioxide followed by autologous hematopoietic cell transplantation for relapsed acute promyelocytic leukemia Blood 2013 121 3095 3102 23412094 10.1182/blood-2012-11-466862

[bib81] Holter Chakrabarty JL Rubinger M Le-Rademacher J Wang HL Grigg A Selby GB Autologous is superior to allogeneic hematopoietic cell transplantation for acute promyelocytic leukemia in second complete remission Biol Blood Marrow Transplant 2014 20 1021 1025 24691221 10.1016/j.bbmt.2014.03.025 PMC4097890

[bib82] Mannis GN Logan AC Leavitt AD Yanada M Hwang J Olin RL Delayed hematopoietic recovery after auto-SCT in patients receiving arsenic trioxide-based therapy for acute promyelocytic leukemia: a multi-center analysis Bone Marrow Transplant 2014 50 40 44 25243620 10.1038/bmt.2014.201

[bib83] Micol JB Raffoux E Boissel N Lengline E Canet E Daniel MT Management and treatment results in patients with acute promyelocytic leukaemia (APL) not enrolled in clinical trials Eur J Cancer 2014 50 1159 1168 24440088 10.1016/j.ejca.2013.11.023

[bib84] Lengfelder E Hofmann WK Nolte F Management of elderly patients with acute promyelocytic leukemia: progress and problems Ann Hematol 2013 92 1181 1188 23694997 10.1007/s00277-013-1788-z PMC3734597

[bib85] Park JH Qiao B Panageas KS Schymura MJ Jurcic JG Rosenblat TL Early death rate in acute promyelocytic leukemia remains high despite all-trans retinoic acid Blood 2011 118 1248 1254 21653939 10.1182/blood-2011-04-346437 PMC3790946

[bib86] Iland HJ Seymour JF Wei A Optimal approach for high-risk acute promyelocytic leukemia Curr Opin Hematol 2014 21 102 113 24378704 10.1097/MOH.0000000000000025

[bib87] Altman JK Rademaker A Cull E Weitner BB Ofran Y Rosenblat TL Administration of ATRA to newly diagnosed patients with acute promyelocytic leukemia is delayed contributing to early hemorrhagic death Leuk Res 2013 37 1004 1009 23768930 10.1016/j.leukres.2013.05.007

[bib88] Rashidi A Riley M Goldin TA Sayedian F Bayerl MG Aguilera NS Delay in the administration of all-trans retinoic acid and its effects on early mortality in acute promyelocytic leukemia: final results of a multicentric study in the United States Leuk Res 2014 38 1036 1040 25035073 10.1016/j.leukres.2014.06.011

[bib89] Tallman MS Altman JK How I treat acute promyelocytic leukemia Blood 2009 114 5126 5135 19797519 10.1182/blood-2009-07-216457

[bib90] Grisariu S Spectre G Kalish Y Gatt ME Increased risk of central venous catheter-associated thrombosis in acute promyelocytic leukemia: a single-institution experience Eur J Haematol 2013 90 397 403 23414485 10.1111/ejh.12087

[bib91] Kumana CR Au WY Lee NS Kou M Mak RW Lam CW Systemic availability of arsenic from oral arsenic-trioxide used to treat patients with hematological malignancies Eur J Clin Pharmacol 2002 58 521 526 12451429 10.1007/s00228-002-0514-x

[bib92] Au WY Kumana CR Kou M Mak R Chan GC Lam CW Oral arsenic trioxide in the treatment of relapsed acute promyelocytic leukemia Blood 2003 102 407 408 12814916 10.1182/blood-2003-01-0298

[bib93] Siu CW Au WY Yung C Kumana CR Lau CP Kwong YL Effects of oral arsenic trioxide therapy on QT intervals in patients with acute promyelocytic leukemia: implications for long-term cardiac safety Blood 2006 108 103 106 16514059 10.1182/blood-2006-01-0054

[bib94] Au WY Kumana CR Lee HK Lin SY Liu H Yeung DY Oral arsenic trioxide-based maintenance regimens for first complete remission of acute promyelocytic leukemia: a 10-year follow-up study Blood 2011 118 6535 6543 21998212 10.1182/blood-2011-05-354530

[bib95] Zhu HH Wu DP Jin J Li JY Ma J Wang JX Oral tetra-arsenic tetra-sulfide formula versus intravenous arsenic trioxide as first-line treatment of acute promyelocytic leukemia: a multicenter randomized controlled trial J Clin Oncol 2013 31 4215 4221 24127444 10.1200/JCO.2013.48.8312

[bib96] Zeidan AM Gore SD New strategies in acute promyelocytic leukemia: moving to an entirely oral, chemotherapy-free upfront management approach Clin Cancer Res 2014 20 4985 4993 25274377 10.1158/1078-0432.CCR-13-2725 PMC4882758

[bib97] Takeshita A Shinjo K Naito K Matsui H Sahara N Shigeno K Efficacy of gemtuzumab ozogamicin on ATRA- and arsenic-resistant acute promyelocytic leukemia (APL) cells Leukemia 2005 19 1306 1311 15920495 10.1038/sj.leu.2403807

[bib98] Castaigne S Pautas C Terre C Raffoux E Bordessoule D Bastie JN Effect of gemtuzumab ozogamicin on survival of adult patients with de-novo acute myeloid leukaemia (ALFA-0701): a randomised, open-label, phase 3 study Lancet 2012 379 1508 1516 22482940 10.1016/S0140-6736(12)60485-1

[bib99] Ravandi F Estey EH Appelbaum FR Lo-Coco F Schiffer CA Larson RA Gemtuzumab ozogamicin: time to resurrect J Clin Oncol 2012 30 3921 3923 22987091 10.1200/JCO.2012.43.0132 PMC4874205

[bib100] Stein EM Stein A Walter RB Fathi AT Lancet JE Kovacsovics TJInterim analysis of a phase 1 trial of SGN-CD33A in patients with CD33-positive acute myeloid leukemia (AML). American Society of Hematology Meeting, San Francisco, CA, USA, 2014..

[bib101] Lo-Coco F Cimino G Breccia M Noguera NI Diverio D Finolezzi E Gemtuzumab ozogamicin (Mylotarg) as a single agent for molecularly relapsed acute promyelocytic leukemia Blood 2004 104 1995 1999 15187030 10.1182/blood-2004-04-1550

[bib102] Hashimoto Y Kagechika H Kawachi E Fukasawa H Saito G Shudo K Correlation of differentiation-inducing activity of retinoids on human leukemia cell lines HL-60 and NB4 J Cancer Res Clin Oncol 1995 121 696 698 7593136 10.1007/BF01218530 PMC12201500

[bib103] Tobita T Takeshita A Kitamura K Ohnishi K Yanagi M Hiraoka A Treatment with a new synthetic retinoid, Am80, of acute promyelocytic leukemia relapsed from complete remission induced by all-trans retinoic acid Blood 1997 90 967 973 9242525

[bib104] Shinagawa K Yanada M Sakura T Ueda Y Sawa M Miyatake J Tamibarotene as maintenance therapy for acute promyelocytic leukemia: results from a randomized controlled trial J Clin Oncol 2014 32 3729 3735 25245439 10.1200/JCO.2013.53.3570

[bib105] Sanford D LoCoco F Sanz MA Di Bona E Coutre S Altman JKSingle agent tamibarotene has activity in acute promyelocytic leukemia patients previously treated with ATRA and arsenic trioxide, but does not produce durable responses. American Society of Hematology Meeting, San Francisco, CA, USA, 2014.

[bib106] Shetty AV Ravandi F Alapati N Borthakur G Garcia-Manero G Kadia TMSurvivorship in APL- outcomes of acute promyelocytic leukemia (APL) patients (pts) after maintaining complete remission (CR) for at least 3 years. American Society of Hematology Meeting, San Francisco, CA, USA, 2014.

